# CT-guided drainage of a brainstem abscess in a cat as an emergency treatment procedure

**DOI:** 10.1177/2055116919896111

**Published:** 2020-02-10

**Authors:** Erika Bersan, Thomas Maddox, Gemma Walmsley, Martina Piviani, Rachel Burrow

**Affiliations:** 1 Small Animal Teaching Hospital, Institute of Veterinary Sciences, University of Liverpool, Neston, UK; 2 Department of Musculoskeletal Biology, Institute of Ageing and Chronic Disease, University of Liverpool, Liverpool, UK

**Keywords:** Brainstem abscess, brain abscess, intracranial abscess, computed tomography (CT)-guided drainage, medical treatment, bacterial meningitis, meningoencephalitis, CT, MRI

## Abstract

**Case summary:**

A 3-year-old male neutered domestic shorthair cat was presented with a 1-week progressive and rapidly deteriorating history of lethargy and abnormal behaviour. Neurolocalisation indicated multifocal intracranial lesions (right oculomotor nerve, brainstem [obtundation, non-ambulatory tetraparesis, vestibular dysfunction and intermittent decerebrate rigidity] and possibly the thalamus [left-sided pleurothotonus]), or more likely a single brainstem lesion with mass effect. MRI of the brain demonstrated a brainstem abscess causing severe dorsal displacement particularly affecting the pons and the medulla oblongata causing cerebellar vermis herniation through the foramen magnum. CT-guided free-hand technique drainage of the brain abscess was performed and broad spectrum antibiotics were started based on sensitivity results. The cat recovered uneventfully from anaesthesia displaying marked improvement immediately after the procedure. Antibiotics were continued for 8 months; repeat imaging prior to withdrawal found complete resolution of the brainstem abscess.

**Relevance and novel information:**

Free-hand CT-guided drainage of a brainstem abscess is not without risk; however, in this case it led to significant clinical improvement and stabilisation likely owing to reduced intracranial pressure. It also provided a diagnostic sample that allowed successful medical treatment planning and outcome. To our knowledge, this is the first report describing the successful management of a brainstem abscess by CT-guided drainage in the veterinary literature. It suggests that stereotactic drainage followed by medical therapy can be considered a successful therapeutic alternative to brain surgery or medical treatment alone, providing an emergency treatment in cases of acute brainstem dysfunction.

## Case description

A 3-year-old, male neutered 4.79 kg, domestic shorthair cat was presented with a 1-week progressive and rapidly deteriorating history of lethargy, abnormal behaviour, inappetence and weight loss (1.8 kg). The cat had been treated by the referring veterinary surgeon for an abscess in the neck region 1 month prior to referral with 1 week oral treatment with amoxicillin and clavulanic acid.

A general physical examination on arrival revealed bradycardia (mean heart rate 100 beats per min [bpm]) and obtundation. The cat was normothermic (38.4°C). On examination the mean systemic blood pressure was 176/116 mmHg. No lesions were noted in the cervical region or oropharyngeal cavity, and the rest of the clinical examination, including otoscopic examination, was unremarkable.

Neurological examination found obtundation, non-ambulatory tetraparesis with left-sided pleurothotonus and intermittent decerebrate rigidity. The postural responses were reduced in all four limbs and were worse on the right side. Cranial nerve evaluation showed an absent menace response and reduced facial sensation on the right, vertical positional nystagmus, anisocoria (right-sided mydriasis) and an absent pupillary constriction on the right in response to direct and indirect stimulation of the pupillary light reflex. Neurolocalisation indicated multifocal intracranial lesions involving the oculomotor nerve on the right (mydriasis unresponsive to light), the brainstem (obtundation, tetraparesis, vestibular dysfunction and decerebrate rigidity) and possibly the thalamus (pleurothotonus), or more likely a single brainstem lesion with mass effect. The differential diagnosis included inflammatory/infectious aetiologies (immune-mediated disorders or bacterial, viral, *Toxoplasma gondii* or parasitic infection) and neoplasia (meningioma, lymphoma).

Haematology revealed a mature neutrophilia (neutrophils 16 × 10^9^/l; reference interval [RI] 2.5–12.5 × 10^9^/l). The biochemistry profile revealed mild increased alanine aminotransferase of 179 U/l (RI 7–50 U/l). Total thyroxine, venous blood gas analysis, electrolytes and coagulation times were within normal limits. Feline immunodeficiency virus antibody and feline leukaemia virus antigen tests were negative, as was *T gondii* serum IgG/IgM antibody testing. The cat was up to date with routine vaccinations and parasite treatments.

**Figure 1:**
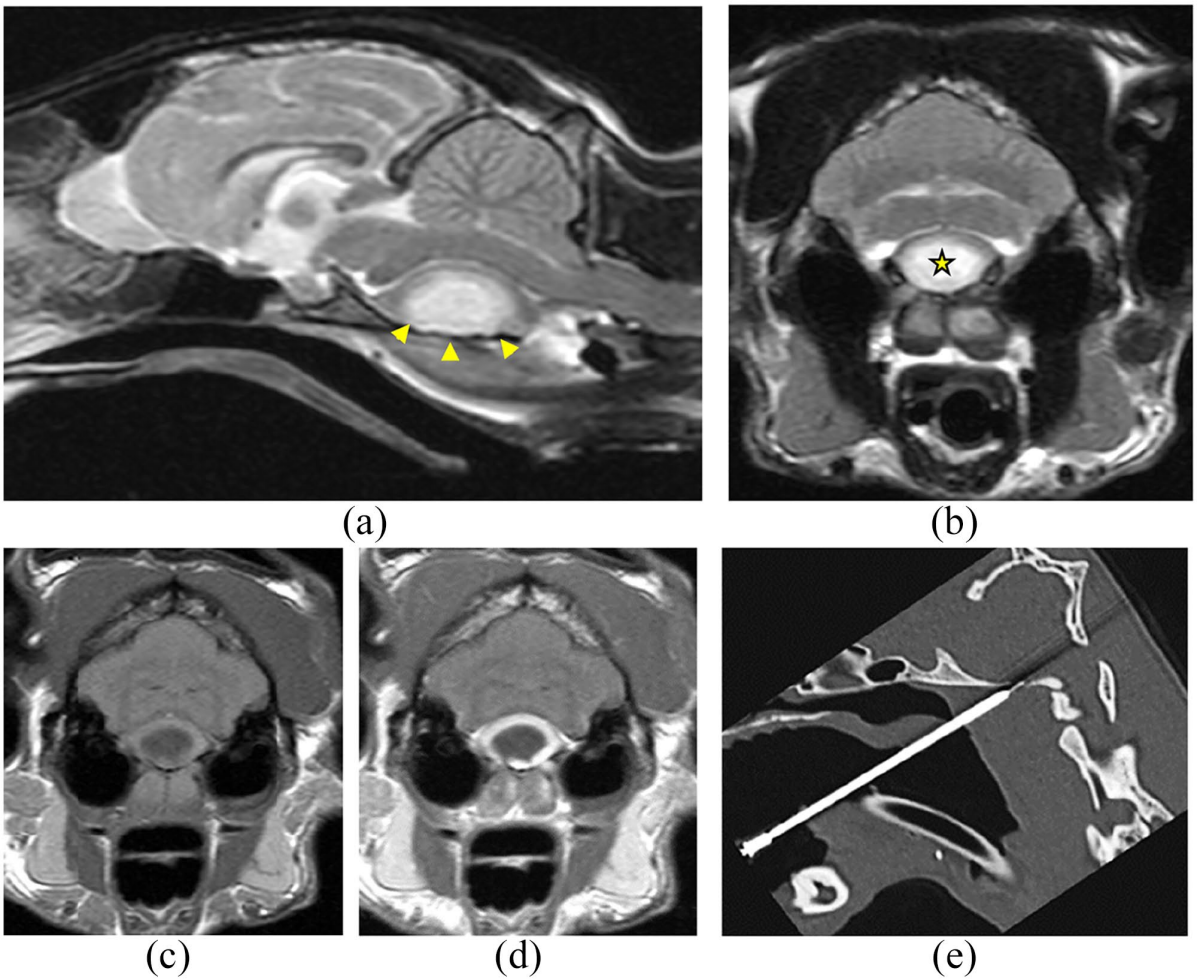
(a) T2-weighted midline sagittal image of the brain; (b) T2-weighted transverse image, (c) T1-weighted transverse and (d) T1-weighted with contrast transverse image at the level of the cerebellar vermis and lateral cerebellar hemisphere. A well-defined, large, extra-axial mass [arrowheads in (a) and star in (b)] causing severe dorsal displacement of the pons and the medulla oblongata and cerebellar vermis herniation through the foramen magnum is visible. (d) A strong and well-defined ring of contrast enhancement is present at its margins. (e) CT mid-sagittal image showing the position of the needle through the soft palate and basioccipital bone

After stabilisation with mannitol (0.5 g/kg [mannitol 20% w/v; B Braun]) administered intravenously on a constant rate infusion over 20 mins and fluid therapy, MRI of the brain was undertaken (Figure 1a–d), under general anaesthesia using a 1.5 Tesla magnet (Philips Ingenia CX). MRI sequences included sagittal, dorsal and transverse T2-weighted (T2W), transverse T2-weighted fluid-attenuated inversion recovery (FLAIR), transverse T2*-weighted gradient echo and transverse three-dimensional volumetric acquisition. Transverse T1-weighted (T1W) sequences were obtained before and after intravenous (IV) administration of gadolinium contrast (gadobutrol 0.1 mmol/ml [Gadovist; Bayer]).

MRI revealed a well-defined, large, extra-axial mass dorsal to the basioccipital bone, which was hypointense on T1W with a mildly hyperintense rim, hyperintense on T2W images and heterogeneously hypointense with a well-defined strongly hyperintense rim on the FLAIR sequence. A strong and well-defined peripheral ring of contrast enhancement was present after administration of paramagnetic contrast agent. The lesion was causing a severe mass effect, with dorsal displacement of neural structures, particularly affecting the pons and the medulla oblongata, with cerebellar vermis herniation through the foramen magnum. There was also T2W hyperintensity and contrast enhancement of the rectus capitis and longus capitis muscles ventral to the basioccipital bone. These MRI findings were most consistent with an intracranial abscess.^1,2^

**Figure 2:**
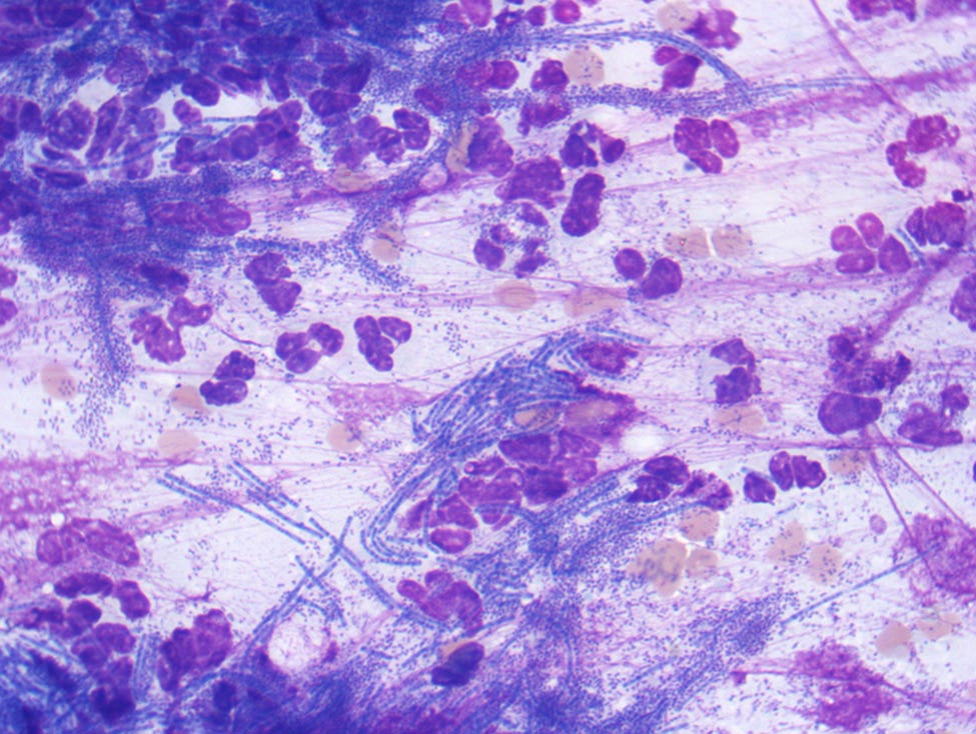
Cytological examination of the fluid-filled brain mass in the cat showing many degenerate neutrophils and many pleomorphic bacteria, including frequent filamentous forms. Wright-Giemsa stain, ×100 objective

Surgical decompression was not considered possible owing to the challenging anatomical location of the lesion. In the light of the severely compromised clinical status of the cat, emergency CT-assisted free-hand drainage of the space-occupying lesion was performed through the soft palate and basioccipital bone (80-slice Toshiba Aquilion CT scanner). A spinal needle (22 G, 2.5”) was used and the procedure was performed under the same general anaesthetic immediately after the MRI scan (Figure 1e). Around 3.8 ml of purulent yellowish fluid material was drained. Cytological examination of this material revealed many degenerate neutrophils and myriads of pleomorphic intracellular (and extracellular) bacteria, including small cocci, rods and also filamentous forms. The cytological interpretation was septic suppurative inflammation due to mixed bacterial infection (Figure 2).

No other abnormalities or foci of infections were found on CT examination of the chest and abdomen.

The cat recovered uneventfully from the anaesthetic and the heart rate normalised (between 140 and 180 bpm), as did the systemic blood pressure (non-invasive blood pressure measurement readings were between 130 mmHg and 140 mmHg). The cat was breathing unaided and able to maintain normal oxygen saturation; partial pressure of oxygen and carbon dioxide levels were within normal limits during and immediately after the procedure. After recovery the cat was able to maintain a sitting position.

Broad spectrum IV antibiotic therapy with metronidazole (10 mg/kg IV q12h [metronidazole 500mg/100 ml; B Braun Melsungen AG]) and cefuroxime (11 mg/kg IV q8h [Zinacef 250 mg; Glaxo Operations UK]) was started after CT drainage of the abscess in addition to fluid therapy at a maintenance rate (compound sodium lactate at 2 ml/kg/h [Hartmann’s Lactated Ringers; B Braun Melsungen AG]). The day after the procedure, the cat’s mental status had dramatically improved – it was more alert, and could walk and eat unaided. The cat was normally hydrated and fluid therapy was stopped. Menace response and pupillary light reflex (PLR) deficits remained unchanged.

Bacteriology results from aerobic culture revealed heavy growth of *Arcanobacterium haemolithicum* and moderate growth of the Gram-positive *Actinomyces pyogenes/Trueperella pyogenes.* The anaerobic culture revealed mixed growth of pleomorphic, mainly Gram-negative rods, identified as *Clostridium hastiforme*.

The cat continued to improve and was discharged 2 days after the procedure on oral antimicrobial treatment, which was continued according to the sensitivity results for 8 months (metronidazole at 10 mg/kg PO q12h [metronidazole 25 mg; Summit Veterinary Pharmaceutical] and cephalexin at 11.4 mg/kg PO q8h [Cephacare Flavour 50 mg; Animalcare]).

The cat was seen monthly for re-examination and remained stable. Four months after diagnosis, cephalexin was stopped owing to the development of gastrointestinal side effects and replaced by amoxicillin-clavulanate (20 mg/kg q12h [Synulox Palatable Tablets 50 mg; Zoetis UK].

**Figure 3:**
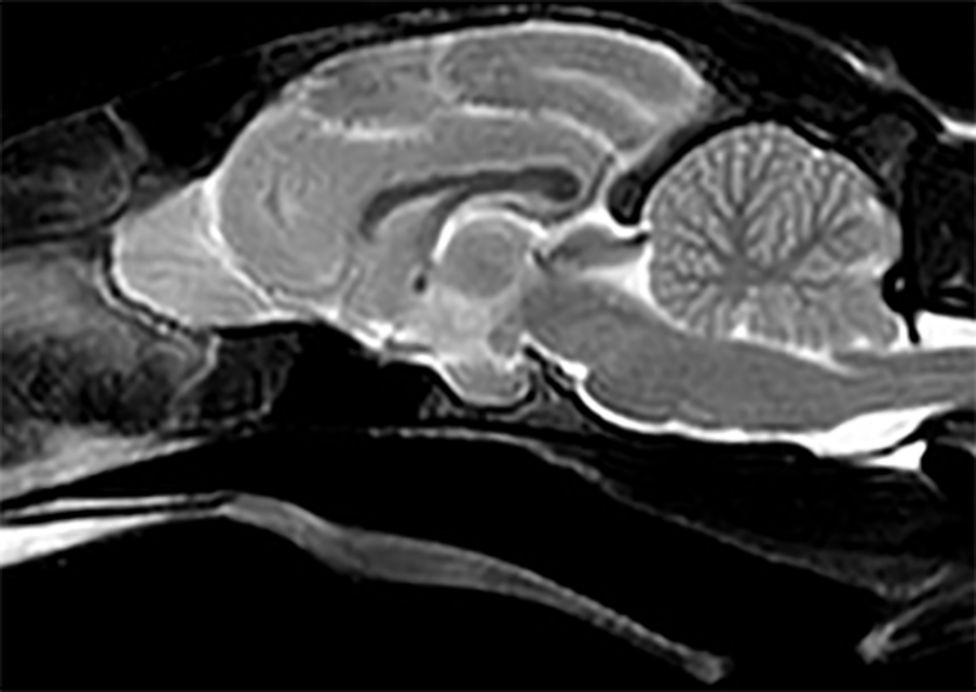
T2-weighted mid-line sagittal image of the brain of the same cat 8 months after diagnosis. Follow-up MRI was performed before withdrawal of antibiotic therapy. Note the complete resolution of the space-occupying lesion present at the time of diagnosis (see Figure 1a)

Repeat imaging performed 8 months after diagnosis and prior to the withdrawal of treatment found complete resolution of the space-occupying lesion (Figure 3). The neurological examination performed before the procedure revealed normal mental status and gait. Intermittent postural reaction deficits could be detected on the right side. Cranial nerve examination found a normal menace response bilaterally and anisocoria with mydriasis on the right, mildly reduced direct and consensual PLR on the right and normal facial sensation. Follow-up examination 16 months after diagnosis found only mild and intermittent right-sided postural reaction deficits that did not affect quality of life. The cat was able to jump and climb, and had returned to previous activity levels with freedom outdoors.

## Discussion

Here we describe the diagnosis and treatment of a ventral brainstem abscess in a cat; to our knowledge, this is the first veterinary report to document the successful management of a brain abscess after CT-guided drainage followed by specific antimicrobial therapy based on sensitivity results.

Treatment of brain abscesses is challenging in both veterinary and human medicine, and requires a multimodal approach including surgical and medical management.^3–9^ Medical treatment combined with surgical decompression (rostrotentorial craniectomy) has been described in eight cases of feline brain abscess attributed to bite wounds and in two canine cases, leading to successful recovery in all but two of the feline cases.^2–4^ Successful medical management alone has also been recently reported with a successful outcome in cases of subdural intracranial empyema and intracranial complication of otitis media/interna (OM/OI; in association with ventral bullae osteotomy) in the veterinary literature.^10–13^

In this case, the unstable clinical signs of the patient and the anatomical location of the abscess would have made surgical exploration challenging and high risk – we therefore attempted CT-guided drainage of the abscess, which was successful. Minimally invasive procedures, such as stereotactic drainage by CT guidance followed by medical treatment, are considered a valuable therapeutic modality for brain abscesses and are often the intervention of choice in human medicine. This is particularly the case for deep-seated abscesses or those located in regions such as the thalamus, basal nuclei or the brainstem, as in this case.^5–8,14,15^ Reoccurrence of abscessation after drainage has been reported in human patients, but this is uncommon if the diameter of the abscess after aspiration has reduced to 1.7–3.4 cm.^5–7,14,15^ In human patients with intracranial abscesses, repeated follow-up imaging is recommended at 1, 2, 3, 6 and 12 months following surgery or CT-guided drainage;^5,6^ however, owing to financial considerations, this is often an unrealistic expectation in veterinary medicine. In this case, neurological re-evaluation was performed monthly and a follow-up MRI scan was carried out at 8 months after the start of treatment; further imaging was offered at 3 and 6 months following diagnosis and drainage, but was declined by the owner.

Recommendations regarding the length of the antimicrobial therapy following drainage vary between the human and veterinary medical literature. In human patients, IV antimicrobials are recommended for between 2 and 8 weeks followed by a 3–6 month course of oral medication. In general, the length of the antimicrobial treatment depends on the immunological status of the patient, the therapeutic response and neuroimaging findings.^5,6,9,14,16^

Corticosteroids are not routinely used in humans with brain abscesses unless severe abscess-related oedema is present. The use of short-term anti-inflammatory doses of steroids is recommended in people with more diffuse forms of bacterial meningitis/meningoencephalitis.^8,17^ Experimental study results in rats suggested that the use of corticosteroids reduces the penetration of the antibiotic within the abscess.^9,18^ The use of a non-steroidal anti-inflammatory drug (meloxicam 0.05 mg/kg PO q24h [Metacam 0.5 mg/ml; Boehringer Ingelheim]) was chosen in this case and continued for 5 days after the procedure.

Brain abscesses may form as a result of haematogenous dissemination of bacteria from a different infectious site or via local spread from an adjacent infection site. The latter includes otogenic abscesses secondary to chronic OM/OI, spread from the retrobulbar region and sinuses, intracranial inoculation from a bite wound or penetrating traumatic injury, migrating foreign body or iatrogenic following brain surgery.^1,2,10,14,15^ A bite wound with the initial insult in the region of the neck and subsequent fistulisation or an otogenic intracranial abscess secondary to chronic OM/OI are the likely ways of entry in this case. No signs consistent with otitis were reported and the inner ear and the external ear canal were normal in the imaging study; however, a small linear amount of amorphous material (T2W hypointense to the grey matter and not contrast-enhancing) was present at the level of the ventral aspect of the right tympanic bulla, which was considered unlikely to be significant in this case. In accordance with this, *A haemolithicum and A pyogenes* are most commonly associated with upper respiratory, and skin and soft tissue infections, including chronic ulceration, wound infection and soft tissue abscessations. *C hastiforme* has not been described in association with brain abscessation in the veterinary literature; however, it has been associated with a brain abscess following a chronic OM in a child from Uganda.^19^

Brainstem abscesses associated with severe clinical signs are rarely reported in the veterinary literature and the prognosis is usually guarded to poor.^1,11,12^ A mortality rate of 100% was reported by Klopp et al in two cases of brainstem abscessation;^1^ intracranial empyema appears to have a more favourable prognosis with a mortality rate of 50% in cases managed with medical treatment alone.^11^ This might be related to the less dramatic, more diffuse and chronic compression associated with empyema vs the more severe and acute increased intracranial pressure and acute neurological dysfunction associated with abscessation in this region. A favourable response to medical treatment (antibiotic and ventral bulla osteotomy) has been reported in a cat with brainstem abscess and active OM/OI; however, no information on the neurological dysfunction or MRI characteristics of the lesion were provided in this case.^13^

## Conclusions

CT-guided drainage of a brainstem abscess is not without risk; however, in this case, it reduced the volume of the mass leading to a dramatic and rapid improvement in clinical signs and provided a diagnostic sample that allowed successful therapeutic planning and an excellent outcome. This case report suggests that stereotactic drainage followed by medical therapy can be considered a successful therapeutic alternative to brain surgery or medical treatment alone in cats with brain abscesses, providing an emergency treatment in cases of acute brainstem dysfunction.
